# Prevalence of Apical Periodontitis in patients with Multiple Myeloma

**DOI:** 10.4317/medoral.23416

**Published:** 2020-03-06

**Authors:** Ricardo E. Oñate-Sánchez, Sara Pérez-Díaz, Francisco J. Rodríguez-Lozano, Julia Guerrero-Gironés

**Affiliations:** 1 Special Care Dentistry and Gerodontology Unit, School of Medicine and Dentistry, IMIB-Arrixaca, University of Murcia, Murcia, Spain

## Abstract

**Background:**

Aim Previous reports have been analyzed the prevalence/association of apical periodontitis (AP) with systemic diseases. The present study aims to analyze the prevalence of healthy/diseased periapex and endodontic treatments in patients with Multiple Myeloma (MM) and compare the results with those of control subjects.

**Material and Methods:**

Methodology Panoramic radiographs of 50 individuals with MM were evaluated and compared with 50 controls that were sex and age matched exactly with the diseased group. Radiographic analysis was performed by 2 two experienced endodontists under standardized conditions. The periapical status (presence or not of AP) was assessed using the periapical index (PAI). Data included systemic health, technical quality of root fillings, total number of teeth, quality of restoration, and periapical status. Statistical evaluation of differences between groups included used chi-squared tests and Fisher’s exact tests.

**Results:**

The prevalence of root canal-treated teeth was 10.11% in the MM group and 12.05% in the control group (*p*=0.90). The average root canal-treated teeth in the test group was 2,34 and 2.48 in the control group, where the difference was statistically significant (*p*=0.05). AP in 1 or more teeth was found in 86 % and in 78% of the patients in the MM and the control groups, respectively. When analyzed by subject, there was no statistically significant difference in the prevalence of AP (*p*>0.72). Similarly there was also no statistically significant difference in the prevalence of PA (*p*=0.85), when analyzed by tooth, AP was found in 63.2% and 62.9% in MM and control groups.

**Conclusions:**

The presence of AP and endodontic treatment was not significantly different in individuals with MM compared with control subjects. Future studies are needed to elucidate and confirm the association between MM and AP.

** Key words:**Apical periodontitis, multiple myeloma, endodontics, root canal treatment, systemic disease.

## Introduction

Apical periodontitis (AP) is the inflammation and destruction of the periapical tissues as a consequence of a pulpal necrosis. It can also result from a pre-existing acute abscess or inadequate endodontic therapy (1,2). AP is a fairly frequent condition that increases with age. It occurs in 34 to 61% of people and 2.8 to 4.2% of teeth in Europe (3). The AP treatment of choice is root canal treatment (RCT), which is estimated at around 41-59% of individuals and 2-6,4% of teeth, with radiographic evidence of persistent chronic AP in 24-65% of root filled teeth (4). Factors like nutrition or the existence of systemic pathology could influence the repair of periapical lesions. Compromised immune systems may play an important role in the development, progression and healing of AP (5). When dental and medical scientific communities have analyzed the relation between AP and systemic health (6), the results suggest an association between PA and diabetes mellitus (7), coronary heart disease (8), tobacco smoking (9) and other diseases like respiratory diseases (10) or osteoporosis in post menopause women (11).

Multiple myeloma (MM) accounts for 10% of neoplasms in bone marrow, the average age of individuals at the time of diagnosis being 65 years, with less than 15% of cases occurring below 50 years. Each year 3-5 cases per 100,000 people are diagnosed (12). The disease is primarily characterized by anemia, increased risk of infection, hypercalcemia, bone lesions and renal failure and it is the second most common hematological malignancy (13). MM interferes with the mechanisms of bone renewal causing its decalcification (osteolytic bone lesions) that usually cause intense bone pain and increase the risk of spontaneous fractures. The most common locations affected are the spine, ribs, pelvis and skull. Diagnosis of MM is made by a complete protein analysis in blood and urine, which allows the monoclonal component to be correctly characterized, while a bone marrow aspirate is used to confirm the presence of abnormal plasma cells in the bone marrow. In addition, the detection of bone lesions must be determined by a radiological study of the entire skeleton. The primary clinical manifestation of the disease will be found in the oral cavity in 14% of myeloma patients (14,15). Maxillofacial manifestations of MM appear more frequently in the mandible than in the maxilla with an incidence of 8-15% (16-19) and they include soft-tissue amyloid deposits (20), external dental root resorption (21), hypesthetic or anesthetic sensation of the lower lip (Vicent symptom), pain, gingival enlargement, swelling, tooth loosening, osteolytic lesions and amyloidosis with macroglossia (22,23). However, there have been no studies evaluating the association between MM and AP.

The purpose of this study was to evaluate the presence of apical periodontitis and endodontic treatments in patients with myeloma multiple in comparison with subjects without myeloma multiple.

## Material and Methods

- Data collection

A total of 220 patients with multiple myeloma were primarily included in the experimental group. These patients were referred to the School of Medicine and Dentistry (Universidad de Murcia, Spain) for an assessment of their oral health status during the years 2013–2015 . Patients were selected if they fulfilled the following inclusion criteria: 1) at least 18 years old; 2) having a complete medical and dental history, including panoramic radiographs of the maxilla and mandible; 3) the presence of at least one root canal treatment; 4) a follow-up radiograph taken a minimum of 1 year after the root canal filling. The following were used as exclusion criteria: 1) patients who refused to have an X-ray examination; 2) patients with removed pieces that were previously endodontically treated at the moment of this study; and 3) patients with other malign or hematological diseases. The University of Murcia Committee on Research Involving Human Subjects of (ID: 1700/2017) approved the study, which was carried out in accordance with the principles outlined in the Helsinki Declaration i (as revised in 2000) on experimentation involving humans. Study subjects and control patients were asked to voluntarily participate in the study and gave their informed written consent.

Following inclusion/exclusion criteria, the experimental group finally comprised 50 individuals affected by multiple myeloma and the presence of root canal treatment. The controls were sex and age matched with the test group so that the same numbers of individuals without MM were included.

- Assessment of radiographs

Panoramic radiographs were taken of all the patients using Vatech Pax-400 (Vatech Co., Ltd., Gyeonggi-do, Korea) by an experienced radiographic technician. Radiographic analysis was performed by two experienced endodontists, who recorded and evaluated the following information for each patient: sex, age, number of teeth present, number and location of teeth endodontically treated, quality of root canal fillings, quality of coronal restoration, length of root canal filling and periapical status. The two examiners were calibrated based on the criteria and variants established before their evaluation.

The results of all of teeth evaluations were recorded, and were categorized as root-filled teeth if they had been filled with radiopaque material in the root canal(s). The periapical status (presence of AP) was assessed using the periapical index (PAI). The roots were categorized as follows: 1, normal periapical structures; 2, small changes in bone structure: 3, changes in bone structure with some mineral loss; 4, periodontitis with a well-defined radiolucent area; and 5, severe periodontitis with exacerbating features. A score greater than 2 (PAI ≥3) was considered to be a sign of periapical pathology. The worst score of all roots was taken to represent the PAI score for multirooted teeth.

Root canal fillings were judged adequate when all canals were obturated with no voids present and root fillings ended from 0-2 mm short of the radiographic apex. Multirooted teeth were categorized by the root of the most defective filling. Coronal restoration was classified as adequate when the restoration was radiographically intact and had no signs of overhangs, open margins or recurrent caries. Discrepant cases were resolved by joint discussion until consensus was achieved.

- Statistical analysis

All analyses were performed in an SPSS environment (Version 22.0; Inc., Chicago, IL, USA). The study of the outcomes was reported as frequency/percentage and compared between the groups using the Pearson chi-square test and Fisher’s exact tests. A t test was used to determine whether or not the mean age between groups differed significantly. The level of significance adopted was *p*<0.05.

## Results

Fifty patients (29 women and 21 men) with MM ranging in age from 35 to 84 years old (63.3810.585) were identified as eligible for this study (Table 1). The control group consisted of 50 subjects (28 women and 22 men) ranging from 38 to 84 years old (63.4412.188). The difference was not statistically significant (*p*> 0.05). The average number of teeth per patient in the test group and the control group were 23.14 and 20.58, respectively, where the difference being statistically significant (*p*=0.02). The prevalence of root canal-treated teeth was 10.11% in the MM group and 12.05% in the control group (*p*=0.90). The average number of root canal-treated teeth in the test group was 2,34 and 2.48 in control group, the difference being statistically significant (*p*=0.05).

AP in one or more teeth was found in 86 % and 78% of patients in the MM and control groups, respectively. When analyzed by subject, there was no statistically significant difference in the prevalence of AP (*p*> 0.72). Similarly, there was no statistically significant difference in the prevalence of PA (*p*=0.85), when analyzed by tooth (63.2% and 62.9% in MM and control groups, respectively).

In the MM group, 63% of the root canal-treated teeth had AP, and in the control group the percentage was 63% (*p*>0.85).

The number of teeth with adequate endodontic filling in the MM group represented 38% of the root canal-treated teeth, 60% of which had AP (*p*=0,65). In the control individuals, teeth with adequate endodontic filling represented 76% of treated teeth, 65% of which had AP (*p*=0.95).

In the MM and control groups respectively, 40% and 67% of teeth revealed adequate crown restoration, in MM group 69.6% of teeth had AP (*p*=0.19), and in the control group 61% (*p*=0.56).

The number of teeth of adequate length in the MM group was equal to 63% of the root canal-treated teeth, 69% of which had AP (*p*=0.88), while in the control group 67% of root canal-treated teeth had an adequate length but 65% of these had AP.

**Table 1:**
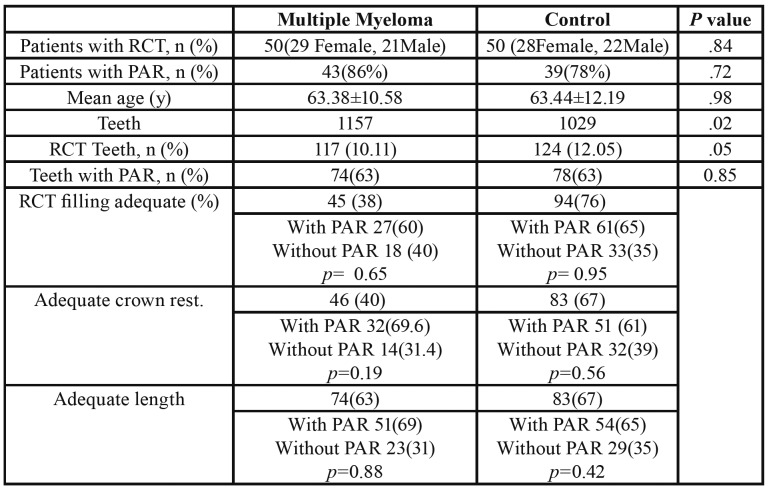
Distribution of the Analyzed Variables in Patients with MM (Study Group) and Control Subjects.

## Discussion

Previous studies have reportedhave reported the strong association between several systemic diseases and endodontic variables, such as apical periodontitis and the outcome of RCT, assessed as root filled teeth (RFT) with radiolucent periapical lesions (RPL) or non-retained RFT (24). The present study aimed to analyze the prevalence of healthy/diseased periapex and endodontic treatments in patients with MM and compare it the same with control subjects (without MM).

Manifestations of Multiple Myeloma include osteolytic lesions or punched-out lesions on the jawbones and it may occur with a similar presentation to other cysts and odontogenic lesions. However, the osteolytic lesions are not usually localized located in periapical regions, being but in the posterior teeth region, ramus, and the mandibular condyle the most frecuent locationscondyle (25).

Regarding to evaluateAs regards evaluating endodontic variables using panoramic radiographs (PAN), this technique is a simple tool in thefor diagnosings of apical periodontitis because of its easy acquisition, low radiation dose, and panoramic views, in agreement withas mentioned by Nardi *et al*. (26). Furthermore, two-dimensional imaging (PAN and periapical radiography) is commonly accepted as a method for the diagnosis of AP, while three-dimensional imaging (cone-beam computed tomographic (CBCT)) remains asrepresents a second- evaluation step evaluation that should be indicated in some cases (26,27).

Due to the immunosuppression status of these patients (28), inclusion and exclusion criteria were strictly applied and patients with other malign or hematological diseases were excluded, in order to reduce the effect of covariables. However, the lack of other studies evaluating the presence of AP lesions and endodontic treatments in MM patients was themust be regarded as the main limitation of this work.

Our data showed that no statistically significant difference was observed between MM and control was observed withas regards to the presence of apical periodontitis and the average size of the periapical lesions (Table 1). However, we observed poor crown restoration and improvable root canal fillings in the Myieloma group. Probably, these data may reflect a lack ofThis may be due to the periodic lack of dental visits due tobeacause they prioritizse theirthe medical problem, or due to the age of these patients. In this respectline, Persic Bukmir *et al*. (29) demonstrated that age was significantly correlated with poor periapical status,. This factorand it has already been defined as a risk indicator for AP (30). Also, thisThe same author confirmed that periodic dental visits to private dental practitionersce reduces the periapical disease ratio , being even more important predictor(29).

Other authors have postulated that the quality of the root canal fillings or the coronal restoration have a similar impact on the resolution of endodontic infections as the influence exerted by an impaired or modulated immune system (5). We didn´tSuch a observe this correlation was not observed in our study, although we think that another probable a limitation might have been the fact was that patients had been suffering from the disease for several years, and not all of them were immunocompromised at the same time. So,Indeed, the severity of the any immunosuppression is another factor that has been reported (6).

## Conclusions

The results of the present study showed that the presence of AP and endodontic treatment was not significantly different in between individuals with MM compared withand control subjects. Furthermore, moreMore studies are needed to elucidate and confirm the association between MM and AP.
